# Transient receptor potential ankyrin 1 (TRPA1) positively regulates imiquimod‐induced, psoriasiform dermal inflammation in mice

**DOI:** 10.1111/jcmm.14392

**Published:** 2019-05-21

**Authors:** Yan Zhou, Dan Han, Taylor Follansbee, Xuesong Wu, Sebastian Yu, Bo Wang, Zhenrui Shi, Dan T. Domocos, Mirela Carstens, Earl Carstens, Samuel T. Hwang

**Affiliations:** ^1^ Department of Dermatology The First Affiliated Hospital of Xi'an Jiaotong University Xi'an Shaanxi China; ^2^ Department of Dermatology University of California Davis California; ^3^ Department of Neurobiology, Physiology and Behavior University of California Davis California; ^4^ Center for Translational Medicine The First Affiliated Hospital of Xi'an Jiaotong University Xi'an Shaanxi China

**Keywords:** imiquimod, inflammation, itch, mice, psoriasis, TRPA1

## Abstract

Transient receptor potential ankyrin 1 (TRPA1), a membrane protein ion channel, is known to mediate itch and pain in skin. The function of TRPA1, however, in psoriasiform dermatitis (PsD) is uncertain. Herein, we found that expression of TRPA1 is highly up‐regulated in human psoriatic lesional skin. To study the role of TRPA1 in PsD, we assessed Psoriasis Severity Index (PSI) scores, transepidermal water loss (TEWL), skin thickness and pathology, and examined dermal inflammatory infiltrates, Th17‐related genes and itch‐related genes in c57BL/6 as wild‐type (WT) and TRPA1 gene knockout (KO) mice following daily application of topical IMQ cream for 5 days. Compared with WT mice, clinical scores, skin thickness change and TEWL scores were similar on day 3, but were significantly decreased on day 5 in IMQ‐treated TRPA1 KO mice (vs WT mice), suggesting reduced inflammation and skin barrier defects. Additionally, the relative area of epidermal Munro's microabscesses and mRNA levels of neutrophil inducible chemokines (S100A8, S100A9 and CXCL1) were decreased in the treated skin of TRPA1 KO mice, suggesting that neutrophil recruitment was impaired in the KO mice. Furthermore, mast cells, CD31^+^ blood vascular cells, CD45^+^ leukocytes and CD3^+^ T cells were all reduced in the treated skin of TRPA1 KO mice. Lastly, mRNA expression levels of IL‐1β, IL‐6, IL‐23, IL‐17A, IL‐17F and IL‐22 were decreased in TRPA1 KO mice. In summary, these results suggest a key role for TRPA1 in psoriasiform inflammation and raising its potential as a target for therapeutic intervention.

## 1 INTRODUCTION

Psoriasis is one of the most common chronic inflammatory skin disorders, affecting about 0.5%‐11.4% of population in the world.^1^ It is characterized by abnormal hyperproliferation of keratinocytes, formation of neutrophilic Munro's microabscesses (MM), dermal angiogenesis and immune cell infiltration.^2^, ^3^ Recently, a growing body of literature implies that the nervous system may be crucial for the pathogenesis of psoriasis. For example, cutaneous denervation resulted in reduction of skin inflammation in psoriasis patients and in a murine model of psoriasiform dermatitis (PsD).^4^, ^5^

The transient receptor potential ankyrin 1 (TRPA1), one member of the large TRP family of ion channels, has emerged as an important regulator of neurogenic inflammation. Other TRP channel proteins such as TRPV1 have been implicated to have regulatory roles in PsD.^6^ TRPA1 is expressed in a subset of C‐fibres that innervate the skin.^7^ Of note, C‐fibre innervation in the epidermis was increased in psoriatic skin.^8^ TRPA1 is also expressed in the keratinocytes, melanocytes, mast cells, as well as other non‐neuronal cells.^9^, ^10^ Additionally, cultured human epidermal keratinocytes produce pro‐inflammatory cytokines such as IL‐1 when treated with a TRPA1 agonist (icilin).^11^ TRPA1 activation by oxazolone resulted in up‐regulation of inflammatory cytokines, including IL‐4, IL‐1β, IL‐16 CXCL‐2, substance P (SP), nerve growth factor (NGF) and endothelin, to facilitate allergic contact dermatitis and pruritus.^12^ In addition to SP and NGF, calcitonin gene‐related peptide (CGRP) has been observed in psoriasis patients with pruritus.^13^ Transient receptor potential ankyrin 1 was recently reported to be involved in histamine‐independent itch.^14^ Therefore, TRPA1, is considered a gatekeeper of cutaneous inflammation and chronic itch. Kemény et al, however, recently suggested that TRPA1 may be a target of imiquimod (IMQ) and that TRPA1 was protective in PsD.^15^ Based on the different conclusions of the studies above, we sought to more conclusively determine if TRPA1 regulates dermal inflammation and barrier function in the IMQ model of PsD that mimics key features of human psoriasis, such as epidermal hyperplasia, MM formation and IL‐17 signalling.^16^ Our findings strongly suggest that TRPA1 positively regulates key clinical, molecular and immunological features of PsD following IMQ treatment for 5 days, rendering it a potential therapeutic target in human disease.

## 2 MATERIALS AND METHODS

### 2.1 Patients

Thirteen psoriasis patients (nine with pruritis: 30.33 ± 7.87 years old, five male patients, four female patients; four without pruritis: 27.75 ± 7.37 years old, two male patients, two female patients) and eight healthy controls (HC) (29.13 ± 8.63 years old, four male patients, two female patients) from The First Hospital of Xi'an Jiaotong University were recruited to the study. All patients with psoriasis were diagnosed according to the criteria based on clinical manifestation and histological examination of psoriasis. Major inclusion criteria were described as follows: no significant infections, immune suppression, hepatic, renal or other diseases; no systemic therapy for at least 4 weeks or topical therapy for at least 2 weeks before enrolling the study. The study followed human ethics protocols in accordance with the principles of the World Medical Association of Helsinki. Studies were approved by The First Hospital Medical Ethics Committee of Xi'an Jiaotong University. Informed consent was obtained from all patients.

### 2.2 Animals

Animal experiments were performed with the procedures that were approved by the University of California Davis Animal Care and Use Committee. Transient receptor potential ankyrin 1 gene knockout mice (KO, −/−, 8‐10 weeks, 20‐25 g) were raised on a C57BL/6 background and were a generous gift of David Julius (University of California San Francisco, USA). Both male and female mice were used.

### 2.3 Psoriasiform dermatitis (PsD) skin inflammation model

The mice were applied to a daily topical dose of 50 mg of IMQ cream (5%) (Aldara, 3M Health Care Limited, UK) on the shaved back (2 cm^2^) for 5 consecutive days. Control mice were treated with same volume of vehicle cream (Vanicream, Pharmaceutical Specialties, Rochester, MN).

### 2.4 Scoring severity of clinical skin inflammation

Psoriasis Severity Index (PSI) score assessment 17 was used to evaluate the psoriatic inflammation in mice on day 3 and day 5 by a blinded observer. PSI‐E (erythema)/PSI‐S (scaling)/PSI‐I (induration) was graded 0 to 4 (0, none; 1, slight; 2, moderate; 3, marked; 4, very marked) and added. The induration (thickness) was further determined by micrometer measurements.

### 2.5 Measurement of dorsal skin thickness

Double‐fold dorsal skin thickness of the treated areas were measured by a blinded observer with a thickness gauge (Peacock, Ozaki, MFG.CO., LTD, Japan) having 0.01 mm accuracy.^18^

### 2.6 Transepidermal water loss (TEWL)

A probe from a Vapo Meter® (Delfin Technologies Ltd., Kuopio, Finland) was applied to the shaved skin for 10 seconds to measure TEWL per manufacturer's recommendation.^19^

### 2.7 Histopathology and immunochemistry (IHC)

Skin tissue samples in formalin‐fixed (10%) were embedded in paraffin for haematoxylin eosin (H&E), IHC and Giemsa staining (for mast cells). We assessed histopathology (epidermal thickness and area of MM) with computer‐assisted quantitative image analysis (Image J, NIH, USA). Tissue sections (5 μm) were prepared for IHC with CD45 (1:400, BD Pharmingen, San Jose, USA), CD3 (1:800, Abcam, San Francisco, USA), F4/80 (1:100, eBioscience, San Diego, USA), CD31 (1:1800, Santa Cruz, Dallas, USA) and TRPA1(1:300, Proteintech, Rosemont, USA). CD3 and mast cells were counted at 200X and expressed as cells/field (200X) in five microscopic fields. CD45 and F4/80 were counted at 400X and expressed as cells/HPF in five microscopic fields.

### 2.8 Quantitative Real‐time PCR (RT‐qPCR)

RNA (extracted from skin specimens with Mini Spin) was converted into cDNA with the high‐capacity first‐strand cDNA Kit (Qiagen). Real‐time PCR was performed with SYBR Green methods on a Step One Plus Realtime PCR system (Applied Biosystem, Carlsbad, CA). Primer for TRPA1(F: 5′‐CCA GGG CGT TGT CTA TGA GG‐3′; R: 5′‐AGG TGT CCA TAT CGT CAC ATC T‐3′) was produced by GENEWIZ, Inc, Suzhou, China. Primers for S100A8 (Mm.PT.58.44003402.gs), S100A9 (Mm.PT.58.41787562), CXCL1 (Mm.PT.58.42076891), CXCL2 (Mm.PT.58.10456839), IL‐17A (Mm.PT.58.6531092), IL‐17F (Mm.PT.58.9739903), IL‐23 (Mm.PT.58.10594618.g), IL‐1β (Mm.PT.58.10005566), IL‐6 (Mm.PT.58.10005566), IL‐22(F: 5′‐CTG CTT CTC ATT GCC CTG TG‐3′; R: 5′‐AGC ATA AAG GTG CGG TTG AC‐3′), NGF (Mm.PT.58.14181538), SP (Mm.PT.58.28422778) and CGRP (Mm.PT.58.30441082) were produced by Integrated DNA Technologies, Inc, Iowa, USA. The relative expression of target genes was confirmed using quantity of target gene/quantity of GAPDH.

### 2.9 Statistical analysis

Results were expressed as mean ± SEM and analyzed statistically using Graphpad Prism 6.02 (Graphpad Software, Inc, San Diego, CA). Student’s *t* test was used to compare the differences between two groups. One‐way ANOVA with post‐hoc comparison (Tukey's HSD) test was used to compare the differences in more than two groups. Two‐way ANOVA followed by Tukey's multiple comparisons test was used to analyze the change in skin thickness and TEWL scores. Statistical significance was defined as *P* < 0.05.

## 3 RESULTS

### 3.1 Increased expression of TRPA1 in skin lesions from psoriasis patients

**Figure 1:**
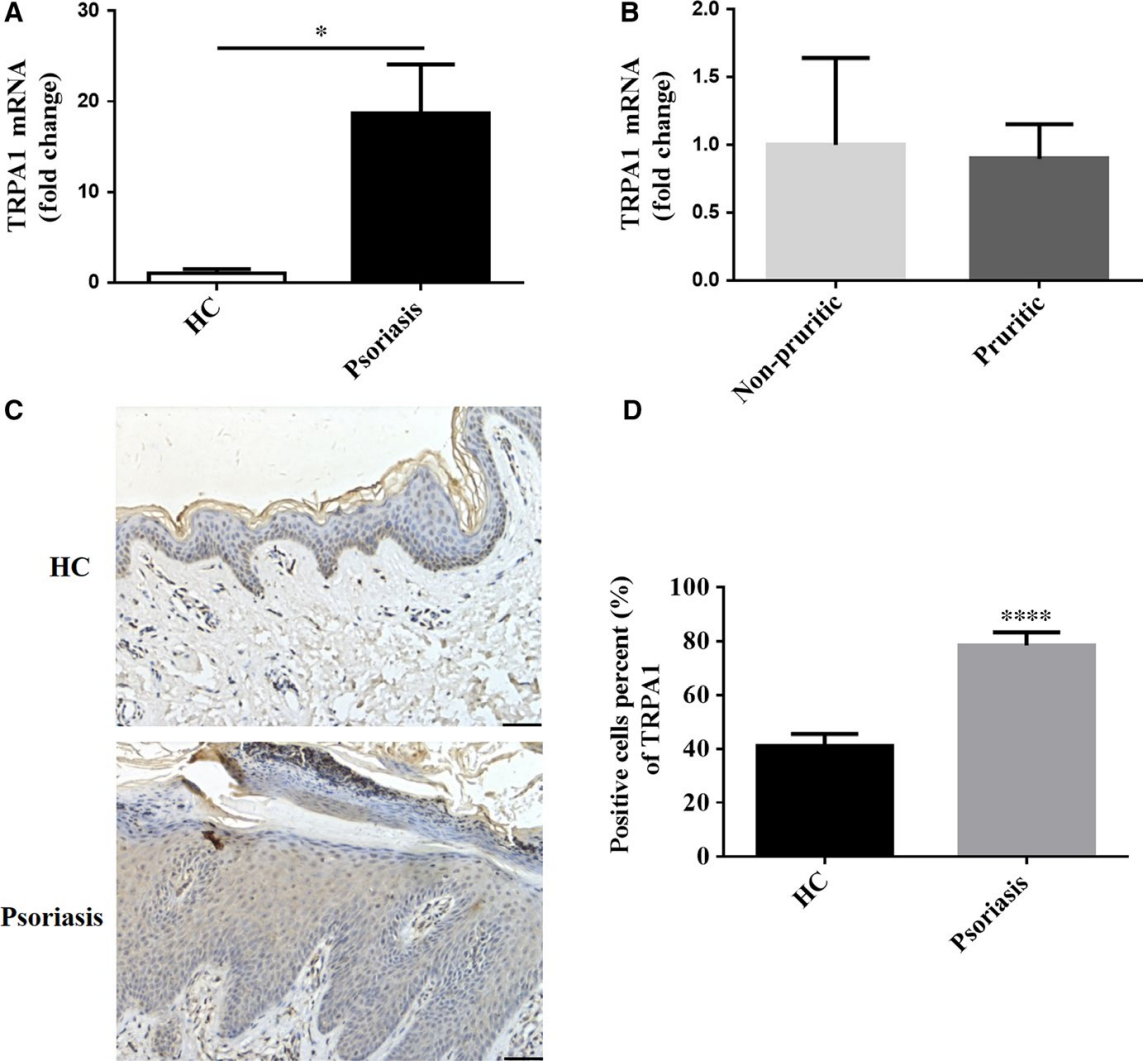
TRPA1 expression is increased in skin lesions from patients with psoriasis. (A) RT‐qPCR analysis of TRPA1 expression in lesional skin from patients with psoriasis (n = 13) and normal skin from HC (n = 8). (B) TRPA1 mRNA expression in lesional skin from pruritic (n = 9) and non‐pruritic (n = 4) psoriasis patients. (C) Representative immunohistochemical images of TRPA1 expression in lesional skin from psoriasis patients and normal skin (Scale bar = 50 μm). (D) Percents of positive‐stained cells of TRPA1 in epidermis from psoriatic psoriasis (n = 13) and HC (n = 8). Results are normalized to GAPDH expression. Values were shown as mean ± SEM. (*P < 0.05, ****P < 0.0001, Student’s *T* test). HC, healthy controls; TRPA1, Transient receptor potential ankyrin 1

Recently, Gene Expression Omnibus (GEO) Profiles have showed that TRPA1 mRNA levels were elevated in human psoriatic skins (ID: 100699076, 100689973). We also found that mRNA expression of TRPA1 was up‐regulated 19‐fold (*P* < 0.05) in the psoriasis lesions compared with HC by RT‐qPCR (Figure 1A). No significant difference of TRPA1 mRNA expression, however, was observed in lesional skin between pruritic and non‐pruritic psoriatic patients (Figure 1B). Transient receptor potential ankyrin 1 was found to be most prominently expressed in neutrophils within MM, keratinocytes of spinous layer and basal layer in epidermis, vascular endothelial cells and inflammatory cells in dermis (Figure 1C). The percentage of positive‐stained cells of TRPA1 was increased in the lesions from the psoriasis patients compared with HC (*P* < 0.0001) (Figure 1D). These results indicate that TRPA1 may participate in psoriasis pathogenesis, but not necessarily in psoriatic itch.

### 3.2 IMQ‐induced PsD is alleviated in TRPA1 KO mice

**Figure 2:**
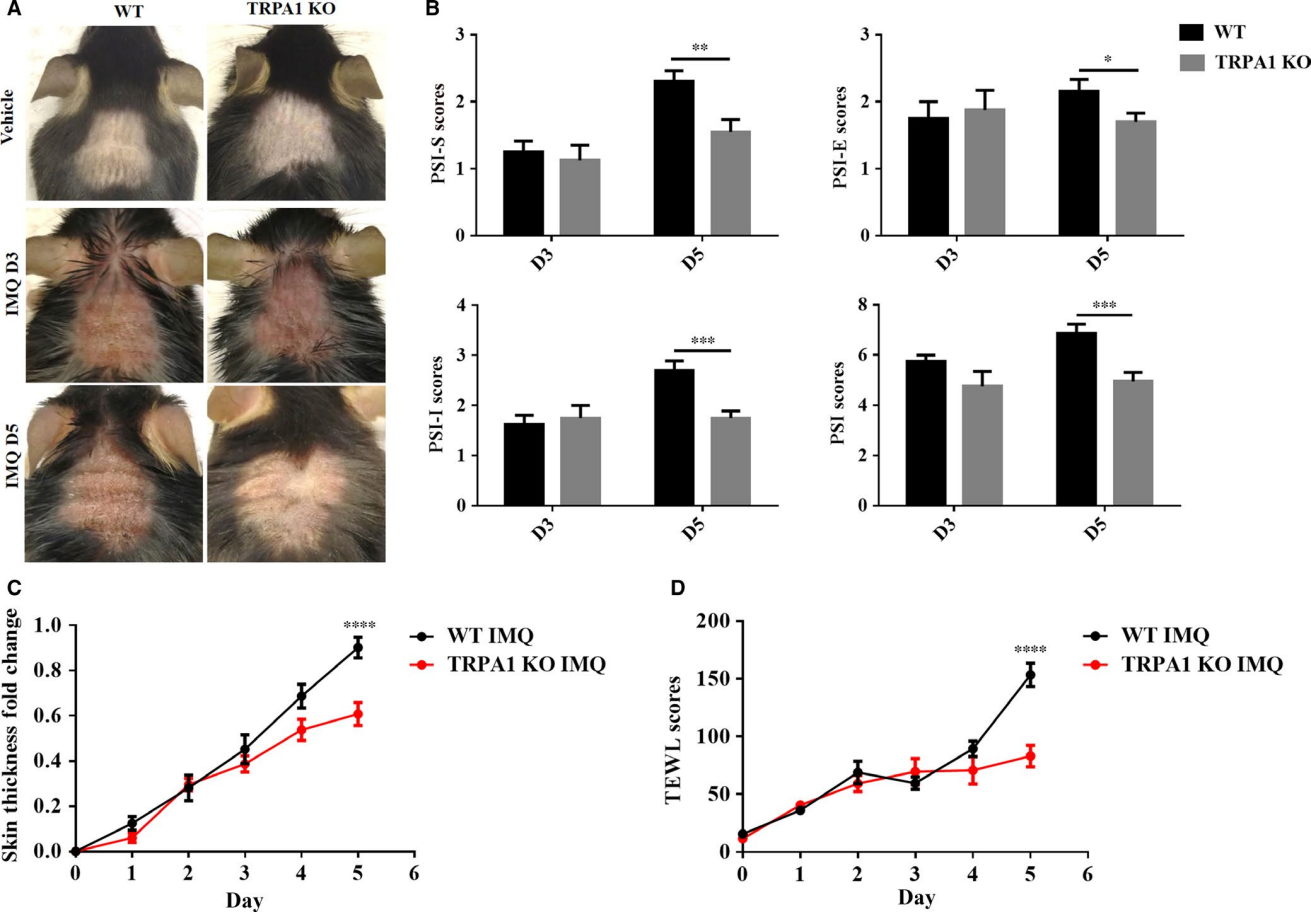
Cutaneous signs are reduced in TRPA1 KO mice following 5 days of treatment with IMQ. (A) Representative images of the dorsal skin after IMQ treatment for 3 days (D3) and 5 days (D5). (B) PSI‐S, PSI‐E, PSI‐I, and total PSI scores in WT and TRPA1 KO mice after IMQ application for 3 days (D3) and 5 days (D5). (C) Fold change in skin thickness. (D) TEWL scores in the skin with IMQ treatment for 5 days. n = 12 in each vehicle group, n = 8‐20 in each IMQ group. PSI scores were assessed by Student’s *T* test; change in skin thickness and TEWL scores by two‐way ANOVA followed by Tukey's multiple comparisons test. Mean ± SEM values are indicated (*P < 0.05, **P < 0.01, ***P < 0.001, ****P < 0.0001). IMQ, imiquimod; KO, knockout; PSI, psoriasiform dermatitis; PSI‐S, PSI‐scaling; PSI‐E, PSI‐erythema; PSI‐I, PSI‐induration; TEWL, transepidermal water loss; TRPA1, Transient receptor potential ankyrin 1

Because of literature suggesting opposing roles for TRPA1 in dermal inflammation,^15^, ^20^ we sought to compare WT and TRPA1 KO mice over the course of a standard 5 days treatment period of IMQ that has been shown to induce PsD in mice. During treatment with topical IMQ, we observed that IMQ‐induced PsD was similar between WT and TRPA1 KO mice on day 3. After an addition 2 days of IMQ, however, signs of psoriasiform inflammation were reduced in the TRPA1 KO group compared with the WT group (Figure 2A). For semi‐quantitative assessment, we assigned a PSI score for each mouse through a blinded observer. At day 5, but not day 3, the total PSI scores of TRPA1 KO mice (*P* < 0.001) were consistently decreased in TRPA1 KO (vs WT) mice, which were reflected in individual scores of scales (PSI‐S) (*P* < 0.01), induration (PSI‐I) (*P* < 0.001) and erythema (PSI‐E) (*P* < 0.05) (Figure 2B). We further measured the double‐fold dorsal skin thickness every day (indicative of dermal inflammation and oedema) and found changes of skin thickness increased in a time‐dependent manner that reached the peak on day 5 (*P* < 0.001). Although no difference was detected after 3 days, dermal induration was reduced by 33% (*P* < 0.0001) after 5 days treatment in TRPA1 KO group compared with WT mice (Figure 2C). Given that TRPA1 KO and WT groups had similar degrees of inflammation at day 3, but that TRPA1 KO mice showed substantially less inflammation at day 5 than WT counterparts, TRPA1 has a positive role in the additional skin inflammation that occurs between day 3 and 5 in this model.

Skin barrier function defects can be measured through a device that objectively and reproducibly quantifies TEWL. Transepidermal water loss has been reported to increase in the lesional skin of psoriasis patients, accompanied by perceived skin dryness and characteristic thick scales.^21^ To objectively measure skin barrier defects in WT and TRPA1 KO mice in the IMQ model, we assessed TEWL throughout the course of IMQ treatment and found that TEWL was markedly decreased in the TRPA1 KO group by 47% (*P* < 0.0001) vs WT mice after treatment of IMQ for 5 days (Figure 2D), implying that TRPA1 is involved in the development of skin dryness and scales in psoriasis and confirming the clinical perception of less severe disease in TRPA1 KO mice. Using multiple clinical measures, we found that TRPA1 KO mice show marked reduced signs of PsD compared to WT mice at a minimum of 5 days of treatment although few if any changes in these two groups of mice could be detected after 3 days.

### 3.3 Decreased epidermal thickness, parakeratosis and dermis angiogenesis in TRPA1 KO vs WT mice treated with IMQ

**Figure 3:**
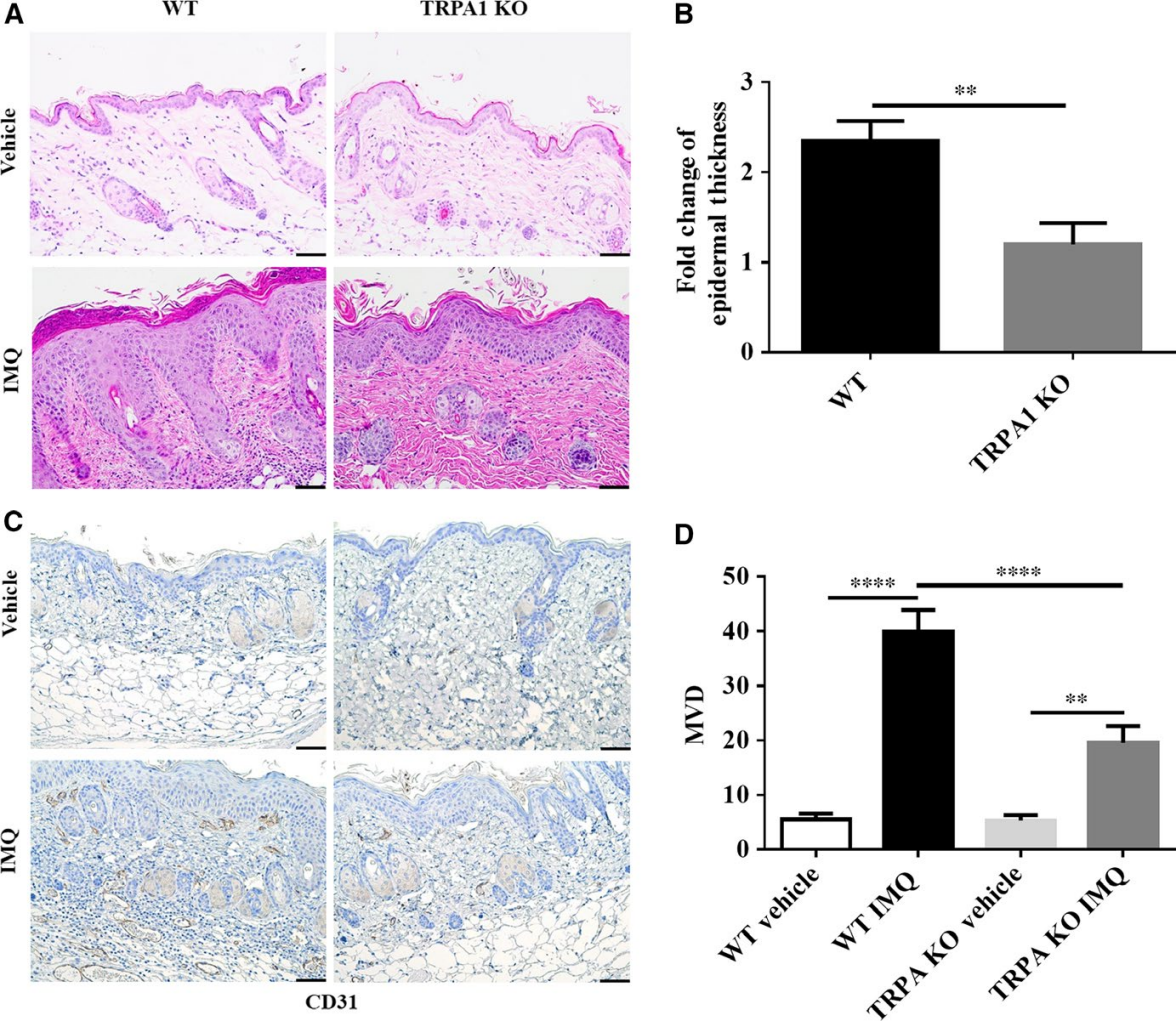
IMQ‐treated TRPA1 KO mice show reduced epidermal thickness and angiogenesis compared to WT mice after 5 days. (A) Histological examination. (B) Fold‐change of epidermal thickness (n = 12 in vehicle group, n = 20 in IMQ group). (C) Representative image of IHC of angiogenesis (CD31). (D) Quantification of MVD (n = 6 per group). Scale bar = 50 μm. *T*‐test for epidermal thickness change, one‐way ANOVA followed by post‐hoc comparison (Tukey's HSD) test for MVD. Mean ± SEM values are indicated (**P < 0.01, ****P < 0.0001). IMQ, imiquimod; KO, knockout; MVD, microvascular density; IHC, Histopathology and immunochemistry; WT, wild‐type

Epidermal hyperplasia and scaling are prominent features of human psoriasis. As expected, hyperkeratosis, parakeratosis and epidermal hyperplasia were present in WT mice but subjectively reduced in TRPA1 KO mice after 5 days of topical IMQ treatment (Figure 3A). Epidermal hyperplasia was decreased in TRPA1 KO mice by 49% (*P* < 0.01) vs WT mice (Figure 3B).

The formation of new blood dilated blood vessels is often seen in human psoriatic skin. We and others have observed morphological changes under microscope such as increases in tortuosity and size of dermal papillary capillaries in the IMQ dermatitis model.^22^ Evaluation of microvascular density (MVD) was performed by staining for the vascular marker, CD31 (Figure 3C). The average MVD for psoriasiform lesions in both WT (*P* < 0.0001) and TRPA1 KO mice (*P* < 0.001) was increased markedly with IMQ treatment, but the extent of this increase in TRPA1 KO mice was decreased by 51% (*P* < 0.001) compared with WT mice (Figure 3D). Thus, along with reduced epidermal hyperplasia, dermis angiogenesis in TRPA1 KO mice is diminished compared to WT mice.

### 3.4 Reduced MM formation and mRNA expression of inflammation‐related genes in TRPA1 KO vs WT mice treated with IMQ

**Figure 4:**
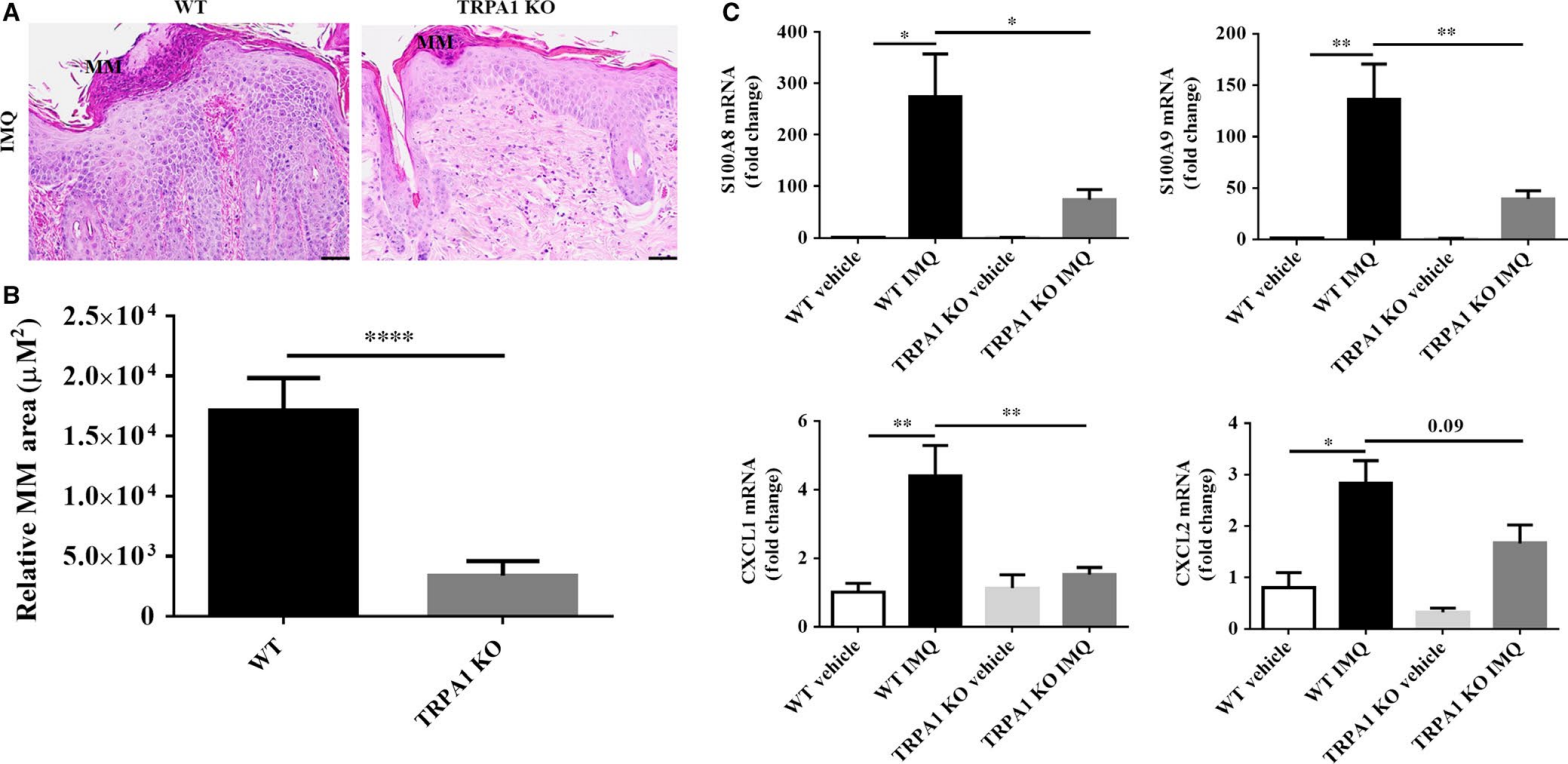
TRPA1 KO mice show diminished neutrophil accumulation and neutrophil‐related cytokines levels following IMQ treatment for 5 days. (A) Representative image of MM (scale bar = 50 μm). (B) Quantification of MM area. (C) RT‐qPCR analysis of S100A8, S100A9 and CXCL1, CXCL2 (the results are normalized to GAPDH expression). n = 12 in each vehicle group, n = 20 in each IMQ group. Student’s *T* test was used for MM area; one‐way ANOVA followed by post‐hoc comparison (Tukey's HSD) test, for mRNA levels. Mean ± SEM values are indicated (*P < 0.05, **P < 0.01, ****P < 0.0001). IMQ, imiquimod; KO, knockout; RT‐qPCR, Real‐time‐qPCR; MM, Munro's microabscesses; TRPA1, Transient receptor potential ankyrin 1

Human psoriatic skin often shows collections of neutrophils in the epidermis and cornified layer known MM. Neutrophils are believed to play a role in psoriasis pathogenesis,^23^ and IMQ‐treated mice skins also feature MM formation.^24^ Munro's microabscesses in IMQ‐treated skin lesion in WT mice were larger and more numerous than those observed in TRPA1 KO mice (Figure 4A). The average area of total MM of each mouse was dramatically diminished in TRPA1 KO mice by 80% (*P* < 0.0001) vs. WT mice (Figure 4B), Congruent with this finding, mRNA levels of neutrophil associated‐proteins S100A8 (*P* < 0.05), S100A9 (*P* < 0.01) and chemokines CXCL1 (*P* < 0.01), CXCL2 (*P* = 0.09) showed varying degrees of reduction in TRPA1 KO mice as compared with IMQ‐treated WT mice (Figure 4C). In aggregate, these results suggest that neutrophils recruitment (a prominent feature of human psoriasis and many murine models of psoriasis) is, in part, regulated by TRPA1 through modulation of expression of neutrophil chemoattractants such as CXCL1 and CXCL2.

### 3.5 Dermal inflammatory cell infiltration in TRPA1 KO vs WT mice treated with IMQ

**Figure 5:**
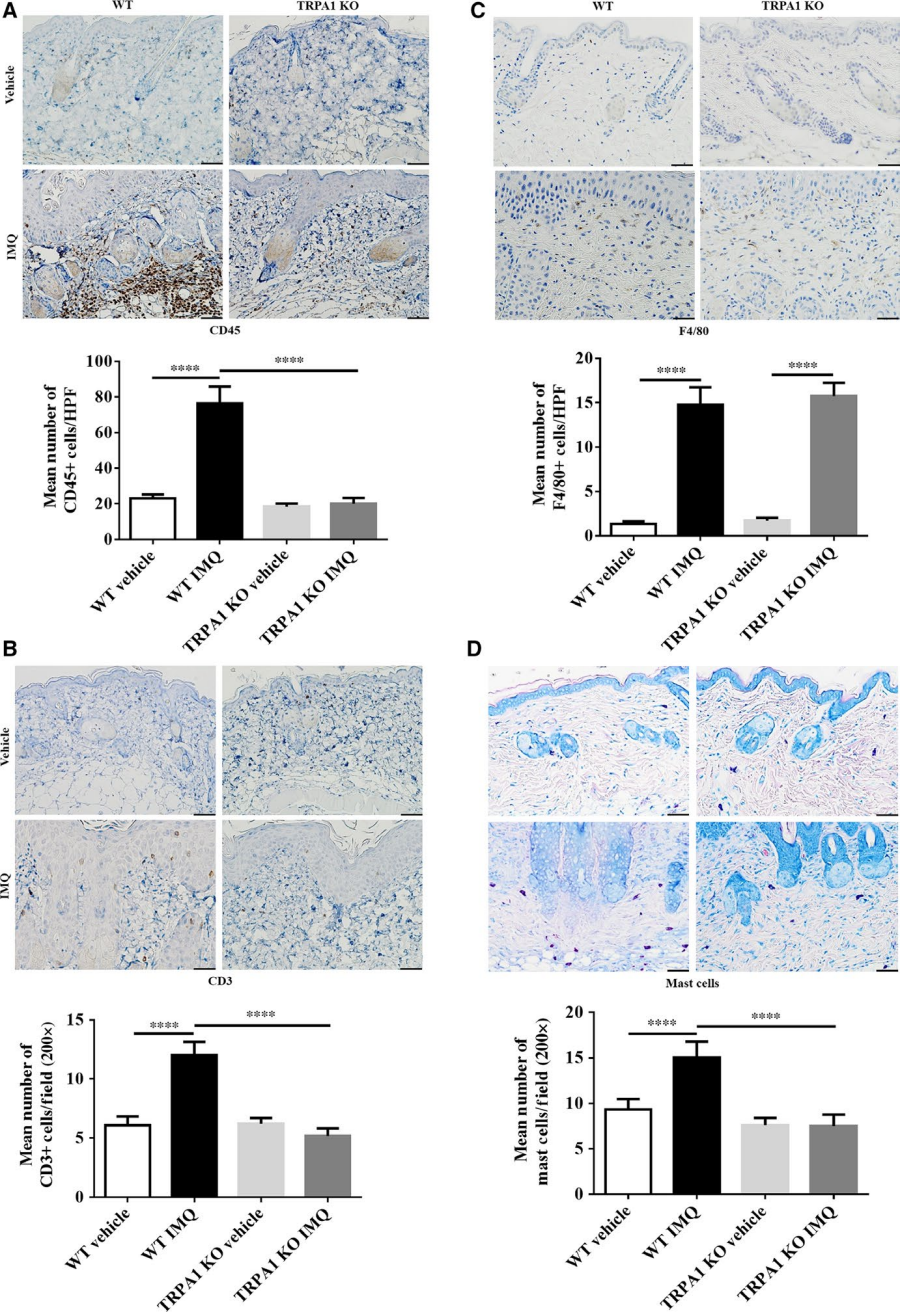
Disruption of TRPA1 decreases infiltration of leukocytes, T cells and mast cells in IMQ‐induced PsD. Representative images of immunohistochemical staining (scale bar = 50 μm) and quantification of (A) CD 45 + leukocytes, (B) F4/80 + macrophages, (C) CD3 + T cells and (D) mast cells. n = 6 per group. All skin lesions were collected after vehicle/IMQ treatment for 5 days. One‐way ANOVA followed by post‐hoc comparison (Tukey's HSD) test was analysed. Mean ± SEM values are indicated (****P < 0.0001). IMQ, imiquimod; TRPA1, Transient receptor potential ankyrin 1

Next, we characterized the dermal inflammatory cell composition in WT and TRPA1 KO mice following daily application of topical IMQ cream for 5 days. We assessed the accumulation of CD45^+^ leukocytes (Figure 5A), CD3^+^ T cells (Figure 5B), F4/80^+^ macrophages (Figure 5C) and mast cells (Figure 5D) in the IMQ‐treated skin of WT and found that the density of these inflammatory cells were all increased in WT mice (*P* < 0.0001). In TRPA1 KO mice, however, the density of leukocytes was reduced overall by 74% (*P* < 0.0001) (Figure 5A). T cells were reduced by 57% (*P* < 0.0001) (Figure 5B). Mast cells were decreased by 50% (*P* < 0.0001) (Figure 5D). Interestingly, macrophage numbers (Figure 5C) were not affected (*P* > 0.05) in the treated skin of TRPA1 KO mice, suggesting that most, but not all, dermal inflammatory cell infiltration is down‐regulated in TRPA1 KO mice.

### 3.6 Reduction of mRNA expression of Th17‐related genes in lesional skin of TRPA1 KO vs WT mice treated with IMQ

**Figure 6:**
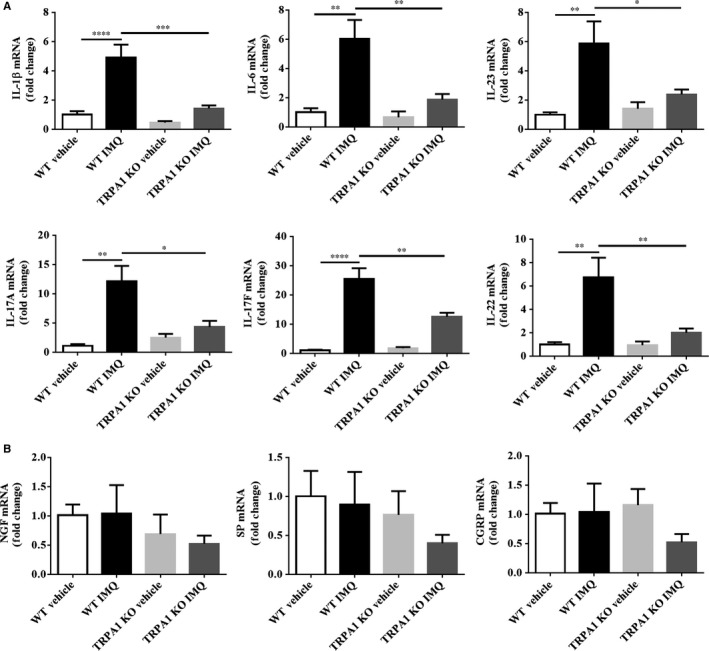
TRPA1 KO mice show reduced mRNA expression of Th17‐related genes in IMQ‐induced PsD skin lesions. (A) RT‐qPCR analysis of IL‐1β, IL‐6, IL‐23, IL‐17A, IL‐17F and IL‐22. (B) RT‐qPCR analysis of NGF, SP and CGRP. These results are normalized to GAPDH expression. n = 12 in each vehicle group, n = 20 in each IMQ group. One‐way ANOVA followed by post‐hoc comparison (Tukey's HSD) test was used. Mean ± SEM values are indicated (*P < 0.05, **P < 0.01, ***P < 0.001, ****P < 0.0001). CGRP, calcitonin gene‐related peptide; IMQ, imiquimod; KO, Knockout; NGF, nerve growth factor; PsD, psoriasiform dermatitis; RT‐qPCR, Real‐time‐Qpcr; SP, substance P; TRPA1, Transient receptor potential ankyrin 1

It has been well established that treatment of mouse skin with IMQ results an up‐regulation of Th17‐related cytokines, but it is unclear how IMQ affects the expression of known itch‐related genes. To determine this, we measured mRNA expression of Th17‐related and itch‐related genes by RT‐qPCR. Of note, Th17‐related inflammatory cytokines such as IL‐1β (*P* < 0.001), IL‐6 (*P* < 0.01), IL‐23 (*P* < 0.05), IL‐17A (*P* < 0.05), IL‐17F (*P* < 0.01) and IL‐22 (*P* < 0.01) in the treated skin of TRPA1 KO mice were all reduced compared with WT mice (Figure 6A). Interestingly, there were no difference in mRNA levels of itch‐related genes (NGF, SP and CGRP) between the two groups (*P* > 0.05) (Figure 6B), substantiating our observation that there was no difference in TRPA1 expression between itchy vs. non‐itchy human psoriatic skin. Thus, TRPA1 regulates key Th17‐related cytokines but does not impact expression of several known itch‐related genes in skin.

## 4 DISCUSSION

Several published reports suggest that TRPA1 could be a key positive regulator of skin inflammation,^20^, ^25^ but others^15^ have suggested that TRPA1 could be protective (at least in murine models of psoriasis). We found TRPA1 mRNA levels increased 19‐fold in psoriatic skin lesions compared with skin from individuals without psoriasis. The percentage of positive‐stained keratinocytes of TRPA1 was increased in the lesions from the psoriasis patients compared with HC. Therefore, we suggest that TRPA1 may indeed play a positive role in psoriasis. Clinical, histological, immunological and molecular studies presented herein show that TRPA1 KO have significantly reduced levels of PsD compared to their WT counterparts.

In comparing our experimental protocol to those of others which suggested that TRPA1 is a negative regulator of inflammation,^15^ we used a relatively higher dose of IMQ and treated 2 days longer. In multiple published studies of IMQ treatment as a model of PsD, 5 or more days of topical treatment with IMQ have been used as a standard endpoint for treatment.^6^, ^16^, ^24^, ^26^ Indeed, in our studies, we did not observe an obvious difference in skin inflammation parameters between WT and TRPA1 KO mice on day 3, suggesting that the role of TRPA1 may be later in the develop of psoriatic changes in this model. Additionally, GEO Profiles have showed that TRPA1 mRNA levels were elevated in mouse dorsal skin after applying a daily topical dose of 62.5 mg IMQ cream (5% Aldara; 3M Pharmaceuticals) for 6 consecutive days (ID: 72253659). This result further supported our observation in humans (Figure 1).

Clinical scoring of psoriatic changes in mouse skin can be somewhat subjective. Therefore, we used an objective a TEWL measurement to measure barrier defects in treated skin. Although TEWL scores are well known to be increased and reflective of barrier defects in atopic dermatitis,^27^ barrier dysfunction as reflected in TEWL measures has also been shown to be present in psoriatic lesions^21^ and can be normalized in psoriasis patients after effective UVB phototherapy^28^ and moisturizers application.^29^ We found TEWL scores reached a peak on day 5 in WT mice with a 46% reduction in TRPA1 KO mice, raising the prospect that TRPA1 may contribute to IMQ‐mediated barrier defects.

Neutrophils play a significant role in psoriasis pathogenesis. Herein, we found that TRPA1 was expressed markedly in neutrophils of MM in epidermis from psoriasis patients. Also, we noticed that the area of MM was markedly decreased in TRPA1 KO vs WT mice, suggesting that neutrophil recruitment is dependent on TRPA1. We also measured mRNA expressions of S100A8, S100A9, CXCL1 and CXCL2 because these genes are expressed by and released from keratinocytes and induce neutrophil immigration.^30^ Relative mRNA levels of these neutrophil inducible cytokines and chemokines were all increased in WT mice treated with IMQ, while the extent of increase was reduced in IMQ‐treated TRPA1 KO mice. It is well known that neutrophils infiltrate the dermis in the initial phase of the psoriatic lesion formation, and consequently they migrate into the epidermis, accumulating in MM.^31^ There is evidence to support the role of TRPA1 in neutrophil infiltration. TRPA1 gene ablation abrogated neutrophil infiltration induced in an acute gout model,^32^ and neutrophil accumulation in the tracheal epithelial was abated by TRPA1 antagonism with acetaminophen administration in mice.^33^ Therefore, we suggest that TRPA1 may contribute to IMQ‐induced dermatitis by mediating neutrophil infiltration through the action of key mediators of neutrophil migration.

It is well known that 50%‐70% of patients with psoriasis exhibit marked pruritus in lesional skin,^13^, ^34^ and the mechanisms underlying itch in psoriasis are just beginning to be understood. We initially suggested that TRPA1, because of it known involvement in some forms of itching, might regular itch in human psoriasis. Interestingly, we found no difference in the expression of TRPA1 mRNA in psoriatic patients with itch compared to those without this symptom, suggesting that the role of TRPA1 in pruritus in psoriasis may be minimal. At the molecular level, our studies confirm that IL‐17 pathway genes are elevated in the IMQ dermatitis model.^16^, ^24^ While these genes are convincingly down‐regulated in TRPA1 KO mice, known genes (NGF, SP and CGRP) that impact itching were not changed. Of note, we found that spontaneous itching behaviour in TRPA1 KO mice and in WT mice treated with IMQ for 5 days were statistically indistinguishable (data not shown), supporting the striking conclusion that reduction of dermal inflammation does not always correspond with decreased itching behaviour in mice within the scope of time used in our experiments. This observation may partly explain why some patients with extensive psoriasis do not show significant pruritus. To a certain extent, these studies imply that inflammation and pruritus are not necessarily identically up‐ or down‐regulated in a temporally coordinated manner.

In summary, TRPA1 plays a clear proinflammatory role in IMQ‐mediated dermal inflammation although it may not be similarly involved in the regulation of itch. Finally, these studies imply that it is a relevant target for pharmacologic intervention in psoriasis and that further study is needed to elucidate the role of TRPA1 in psoriasis.

## CONFLICT OF INTEREST

None.

## AUTHOR CONTRIBUTIONS

Yan Zhou contributed to the experimental design and manuscript writing; Samuel T. Hwang and Earl Carstens conceived and designed the experiments with both theoretical and technical guidance throughout the entire work. Yan Zhou, Dan Han, Taylor Follansbee, Xuesong Wu, Sebastian Yu, Bo Wang, Zhenrui Shi and Dan T. Domocos contributed to conducting experiments and data analysis; Mirela Carstens provided reagents. Samuel T. Hwang and Dan Han participated in manuscript writing and corrected the manuscript. All authors read and approved the final manuscript.

## DATA AVAILABILITY STATEMENT

All data used or analysed during this study are included in this published article.
